# Fluid loading and norepinephrine infusion mask the left ventricular preload decrease induced by pleural effusion

**DOI:** 10.1186/s40635-017-0158-x

**Published:** 2017-09-11

**Authors:** Kristian Borup Wemmelund, Viktor Kromann Ringgård, Simon Tilma Vistisen, Janus Adler Hyldebrandt, Erik Sloth, Peter Juhl-Olsen

**Affiliations:** 1 Department of Anaesthesiology and Intensive Care, Aarhus University Hospital, Palle Juul-Jensens Boulevard 99, 8200 Aarhus N, Denmark; 2 Department of Clinical Medicine, Aarhus University, Palle Juul-Jensens Boulevard 82, 8200 Aarhus N, Denmark; 3 Research Centre of Emergency Medicine, Aarhus University, Nørrebrogade 44, 8000 Aarhus C, Denmark; 4 Division of Medicine, Akershus University Hospital, Lørenskog, Norway; 5 University of Cape Town, Cape Town, South Africa

**Keywords:** Pleural effusion, Animal models, Ventricular function, Fluid therapy, Norepinephrine, Thoracentesis

## Abstract

**Background:**

Pleural effusion (PLE) may lead to low blood pressure and reduced cardiac output. Low blood pressure and reduced cardiac output are often treated with fluid loading and vasopressors. This study aimed to determine the impact of fluid loading and norepinephrine infusion on physiologic determinants of cardiac function obtained by ultrasonography during PLE.

**Methods:**

In this randomised, blinded, controlled laboratory study, 30 piglets (21.9 ± 1.3 kg) had bilateral PLE (75 mL/kg) induced. Subsequently, the piglets were randomised to intervention as follows: fluid loading (80 mL/kg/h for 1.5 h, *n* = 12), norepinephrine infusion (0.01, 0.03, 0.05, 0.1, 0.2 and 0.3 μg/kg/min (15 min each, *n* = 12)) or control (*n* = 6). Main outcome was left ventricular preload measured as left ventricular end-diastolic area. Secondary endpoints included contractility and afterload as well as global measures of circulation. All endpoints were assessed with echocardiography and invasive pressure-flow measurements.

**Results:**

PLE decreased left ventricular end-diastolic area, mean arterial pressure and cardiac output (*p* values < 0.001), but fluid loading (20 mL/kg) and norepinephrine infusion (0.05 μg/kg/min) restored these values (*p* values > 0.05) to baseline. Left ventricular contractility increased with norepinephrine infusion (*p* = 0.002), but was not affected by fluid loading (*p* = 0.903). Afterload increased in both active groups (*p* values > 0.001). Overall, inferior vena cava distensibility remained unchanged during intervention (*p* values ≥ 0.085). Evacuation of PLE caused numerical increases in left ventricular end-diastolic area, but only significantly so in controls (*p* = 0.006).

**Conclusions:**

PLE significantly reduced left ventricular preload. Both fluid and norepinephrine treatment reverted this effect and normalised global haemodynamic parameters. Inferior vena cava distensibility remained unchanged.

The haemodynamic significance of PLE may be underestimated during fluid or norepinephrine administration, potentially masking the presence of PLE.

## Background

A growing number of clinical and experimental studies unambiguously show that pleural effusion (PLE) not only causes respiratory derangement but also may significantly compromise circulation [1–5]. PLE impairs circulation by decreasing left ventricular (LV) preload resulting in hypotension, low cardiac output (CO) and, in the worst cases, shock [6–8].

Patients presenting with low blood pressure or shock are commonly resuscitated with fluid loading, vasopressors and inotropes either as single therapy or in combination [9, 10]. The selected treatment is initiated to increase peripheral resistance or blood flow and, hence, blood pressure. However, blind manipulation of these determinants of blood pressure carries a risk of unphysiological restoration of blood pressure and CO, potentially harming the patient without treating the underlying cause.

We have recently shown that, in addition to the desired inotropic effect, dobutamine aggravated the preload depletion already caused by PLE [11]. PLE is a frequent finding in critically ill patients [12–14], who often present with clinical symptoms similar to, e.g. distributive shock. As fluid loading and norepinephrine are first-line treatments for low blood pressure and shock, the risk of symptomatic and potentially fatal mistreatment is evident. Detailed knowledge of the haemodynamic effects of fluid loading and norepinephrine administration in the presence of PLE is thus crucial for an optimal treatment strategy.

The aim of this study was to examine the effects of fluid loading and norepinephrine administration on invasive measures of global circulation and echocardiographic indices of basic physiologic determinants in a porcine model with PLE. We hypothesised that a PLE-induced reduction in LV preload as measured by LV end-diastolic area (LVEDA) would be restored by fluids and norepinephrine and hence normalise blood pressure and CO.

## Methods

### Animal preparation

Thirty-three female Danish Landrace and Yorkshire piglets (21.9 ± 1.3 kg) were anaesthetised with midazolam 0.5 mg/kg and S-ketamine 0.25 mg/kg. Pentobarbital 10 mg/kg was given before intubation. Anaesthesia was maintained with infusion of fentanyl 10 μg/kg/h and propofol 5 mg/kg/h. The piglets were subject to volume-controlled ventilation (S/5 Datex-Ohmeda Avance, GE HealthCare, Horten, Norway) with a tidal volume of 10–12 mL/kg, oxygen fraction of 0.50 and positive end-expiratory pressure set off maintaining actual expiratory pressures of 2–3 cmH_2_O. End-tidal CO_2_ values were kept between 4 and 6 kPa. Piglets received a continuous fluid infusion of Lactated Ringer’s solution (2 mL/kg/h).

Arterial and venous sheaths were inserted bilaterally using ultrasonographic guidance. A pulmonary artery catheter (Edwards Lifescience, CA, USA) was positioned in the pulmonary artery and a Ventri-Cath catheter (Millar, Inc., Texas, USA) was placed in the left ventricle to measure pressure continuously using a PowerLab station (Millar, Inc., Texas, USA). Continuous ECG and arterial blood pressure were acquired throughout the experiment. Bilateral percutaneous chest tubes were inserted (Portex®, Smiths Medical International Ltd., Minnesota, USA) for pleural fluid installation.

### Study protocol

After stabilisation, a volume of 75 mL/kg temperate vegetable oil was installed equally into both pleural spaces. The piglets then stabilised for 30 min before PLE readings and were randomly assigned in a 2:2:1 allocation ratio into three groups using www.randomization.com: a fluid loading group (*n* = 12) received continuous infusion of Lactated Ringer’s solution (80 mL/kg/h) persisting in six 15-min intervals, a norepinephrine group (*n* = 12) treated with incremental infusion rates in six intervals of 15 min each (0.01, 0.03, 0.05, 0.1, 0.2 and 0.3 μg/kg/min) and a control group (*n* = 6) with no further intervention. PLE was evacuated in all groups, at which point fluid loading was discontinued whereas norepinephrine infusion continued at the maximal infusion rate. The final data point was obtained 30 min after evacuation.

The investigator performing the experiment and obtaining data including echocardiography was blinded to the intervention throughout the experiment and during offline analyses.

### Physiological determinants, data acquisition and analyses

Echocardiography was performed using a Vivid S6 ultrasound system (GE Healthcare, Horten, Norway) equipped with a cardiac M4S probe. Image acquisition of the parasternal long-axis view was performed as described previously [15]. The inferior vena cava was visualised in a long-axis view, and data covering at least one respiratory cycle was captured.

### Preload

**Fig. 1:**
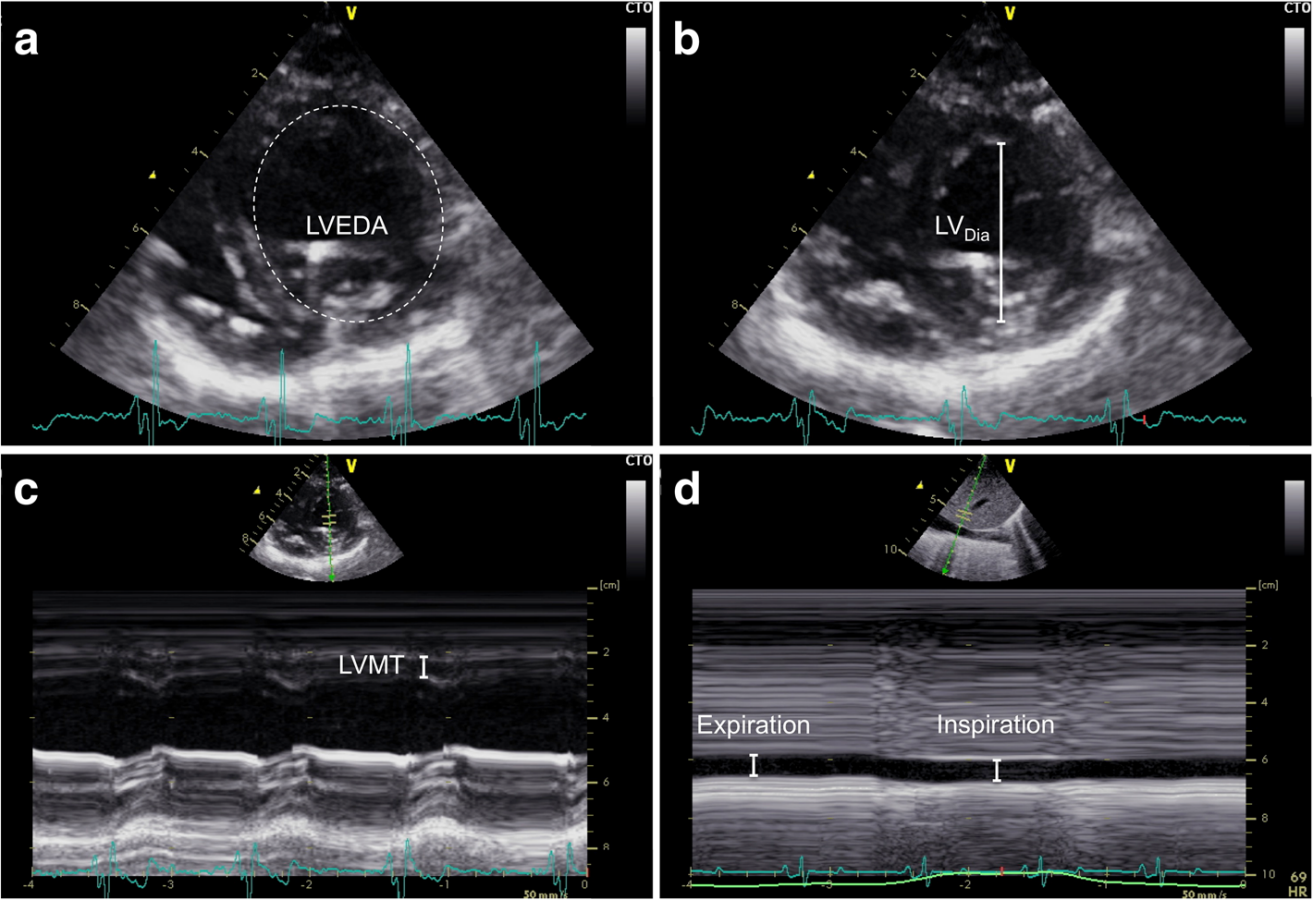
Ultrasonographic data acquisition. Ultrasonographic images of the left ventricle obtained from a parasternal short-axis view. Endocardial tracing allowed for measurement of left ventricular end-diastolic area (LVEDA) (**a**), left ventricular systolic diameter (LV_Dia_) (**b**) and left ventricular myocardial thickness (LVMT) (**c**) to enable afterload calculation. Inferior vena cava diameter was measured with anatomical M-mode at expiration and inspiration (**d**) guided by a respiratory tracing curve (green)

LV preload was estimated as LV end-diastolic area (LVEDA). LVEDA was measured by tracing the LV endocardium at end-diastole, defined as just before the ECG R-wave (Fig. 1a).

### Afterload

Afterload requires both assessment of the LV pressure and LV dimensions measured simultaneously in systole [16]. LV afterload was calculated as:

LV afterload = (LV pressure × LV systolic diameter)/2 × LV myocardial thickness.

LV cavity diameter was obtained from 2-D images (Fig. 1b) and LV myocardial thickness was calibered using anatomical M-mode (Fig. 1c). LV systolic pressure was gathered from the LV catheter. All measures were obtained 100 ms after the ECG S-wave.

### Myocardial contractility

LV myocardial contractility was determined by calculating the LV fractional area change, derived from endocardiac tracings in the same cardiac cycle, as:

LV fractional area change = ((LV end-diastolic area − LV end-systolic area)/LV end-diastolic area) × 100%.

### Inferior vena cava dynamics

The diameters of the IVC were measured with tracings perpendicular to the vessel walls approximately 2–3 cm upstream to the diaphragm (Fig. 1d). The maximal expiratory and inspiratory diameter was measured in the same respiratory cycle with the guidance of a respiration curve automatically generated from respiratory changes in thoracic impedance. The distensibility of the IVC was calculated as:

IVC distensibility = ((inspiratory IVC diameter − expiratory IVC diameter)/expiratory IVC diameter) × 100%.

### Extraction of pulse pressure variation

Pulse pressure variation [17] was derived based on the ECG and arterial blood pressure waveforms as previously described [18]. Briefly, maximal and minimal pulse pressures were calculated for each respiratory cycle of a 1-min window resulting in a series of pulse pressure variation representatives for that minute. The representatives between the 40th and 60th percentile were averaged, removing the effect of arrhythmias.

### General haemodynamic parameters

CO and central venous pressure (CVP) were measured from the pulmonary artery catheter using a Vigilance monitor (Edwards LifeScience, CA, USA). Mean arterial pressure (MAP), heart rate and arterial partial pressure of oxygen (PaO_2_) were obtained via a central arterial line. LV end-diastolic pressure measured immediately before atrial contraction at expiration and was acquired from the LV catheter. Invasive pressures and ECG were stored continuously using S5 Collect software (Datex-Ohmeda, Helsinki, Finland).

LVEDA was the primary endpoint. Secondary endpoints were ultrasonographic indices of LV afterload, contractility and the inferior vena cava along with pulse pressure variations and invasive pressure and flow measures.

### Statistics

A power calculation was based on the difference between two dependent means and standard deviations from a previous study [19]. A 25% increase in LVEDA after a fluid bolus of 20 mL/kg was considered clinically relevant. A total sample size (*β* = 0.9 and *α* = 0.05) was estimated to 12 subjects in each intervention group. For all continuous variables, a mixed model of univariate repeated measurements was used to analyse the within-group time dependence. Paired Student’s *t* test was used to analyse the differences in the same group between two data points. Variables were considered normalised when no statistical difference compared with baseline prior to PLE installation was found. The primary observer performed blinded offline analyses of all data. Subsequently, the primary and a secondary observer performed a blinded reanalysis of 50% of all the echocardiographic measurements. Inter- and intra-observer variation was calculated according to the Bland-Altman principle [20]. Data is presented as mean with the corresponding standard deviation, and a two-sided *p* < 0.05 was considered statistically significant. The STATA software 13.1 (StataCorp LP, College Station, TX, USA) was used throughout.

## Results

A total of 33 piglets (21.9 ± 1.3 kg) were included. Three piglets were excluded due to substantial data loss (*n* = 1), pneumothorax (*n* = 1) and circulatory collapse after PLE installation (*n* = 1), leaving 30 pigs for investigation. Haemodynamic data before and after PLE installation are given in Table 1. Data from the fluid loading group, norepinephrine group and control group are presented in Table 2 (2a–2c), respectively.

**Table 1:** Pleural effusion and haemodynamic variables

	Baseline	Pleural effusion	*p* value
LVEDA (cm^2^)	11.6 ± 1.1	9.8 ± 1.2	0.001
LVESA (cm^2^)	7.3 ± 1.2	5.8 ± 1.0	0.001
IVC_Dia_ (cm)	0.9 ± 0.3	1.0 ± 0.3	0.352
dIVC (%)	5 ± 11	6 ± 9	0.703
CVP (mmHg)	4 ± 3	7 ± 3	0.001
Afterload (mmHg)	90 ± 21	68 ± 17	0.001
LVFAC (%)	37 ± 7	40 ± 9	0.205
MAP (mmHg)	77 ± 12	62 ± 14	0.001
CO (L/min)	2.2 ± 0.5	1.9 ± 0.4	0.001
HR (bpm)	62 ± 8	64 ± 18	0.619
LVEDP (mmHg)	8 ± 3	10 ± 3	0.001
PPV (%)	11 ± 5	11 ± 3	0.976
PaO_2_ (kPa)	32 ± 3	12 ± 4	0.001

Table 1 depicts the haemodynamic variables as mean ± standard deviation at baseline and after 75 mL/kg bilateral pleural effusion installation

*Abbreviations*: *LVEDA* left ventricular end-diastolic area, *LVESA* left ventricular end-systolic area, *IVC*_*Dia*_ expiratory inferior vena cava diameter, *dIVC* inferior vena cava distensibility, *CVP* central venous pressure, *Afterload* left ventricular afterload, *LVFAC* left ventricular fractional area change, *MAP* mean arterial pressure, *CO* cardiac output, *HR* heart rate, *LVEDP* left ventricular end-diastolic pressure, *PPV* pulse pressure variation, *Pa0*_*2*_ arterial partial pressure of oxygen

**Table 2:** The haemodynamic changes of pleural effusion and subsequent fluid loading (2a), increasing infusion rates of norepinephrine (2b) or control (2c)

	Baseline	Pleural effusion							Recovery
2a									
Fluid load (mL/kg)	–	–	20	40	60	80	100	120	120
LVEDA (cm^2^)	11.8 ± 1.1	10.1 ± 1.0^a^	11.4 ± 1.2^b^	11.9 ± 1.4	11.7 ± 1.1	11.9 ± 1.3	11.7 ± 1.5	12.0 ± 1.6^c^	12.4 ± 1.4
LVESA (cm^2^)	7.9 ± 0.9	5.8 ± 0.9^a^	6.4 ± 1.1	6.8 ± 1.0	6.6 ± 1.0	6.8 ± 1.3	6.7 ± 0.9	6.6 ± 0.8^c^	7.3 ± 1.4
IVC_Dia_ (cm)	0.9 ± 0.2	1.0 ± 0.2	1.1 ± 0.2	1.0 ± 0.3	1.1 ± 0.2	1.2 ± 0.2	1.1 ± 0.2	1.1 ± 0.2^c^	1.0 ± 0.1
dIVC (%)	7 ± 7	8 ± 9	6 ± 5	6 ± 5	4 ± 6	8 ± 9	7 ± 5	12 ± 12	9 ± 7
CVP (mmHg)	4 ± 3	8 ± 4^a^	14 ± 8	14 ± 7	14 ± 7	15 ± 7	16 ± 7	12 ± 4^c^	8 ± 4^d^
Afterload (mmHg)	190 ± 44	141 ± 32^a^	189 ± 62^b^	191 ± 65	182 ± 42	182 ± 38	186 ± 36	186 ± 21^c^	188 ± 32
LVFAC (%)	33 ± 5	42 ± 9^a^	43 ± 9	43 ± 7	43 ± 6	43 ± 9	43 ± 5	44 ± 6	40 ± 9^d^
MAP (mmHg)	77 ± 12	65 ± 14^a^	74 ± 16^b^	78 ± 15	78 ± 13	78 ± 12	77 ± 12	78 ± 12^c^	76 ± 21
CO (L/min)	2.3 ± 0.3	2.0 ± 0.3^a^	2.3 ± 0.5^b^	2.7 ± 0.8	2.6 ± 0.6	2.7 ± 0.6	2.4 ± 0.3	2.5 ± 0.3^c^	2.6 ± 0.4
HR (bpm)	64 ± 9	69 ± 25	64 ± 18	66 ± 19	66 ± 16	66 ± 14	64 ± 12	64 ± 13	63 ± 13
LVEDP (mmHg)	8 ± 4	11 ± 3	17 ± 7	17 ± 8	17 ± 4	17 ± 3	18 ± 3	17 ± 3^c^	13 ± 2^d^
PPV (%)	9 ± 1	12 ± 4	9 ± 3^b^	8 ± 3	7 ± 2	6 ± 2	6 ± 2	6 ± 2^c^	7 ± 2
PaO_2_ (kPa)	33 ± 2	12 ± 4^a^	12 ± 4	13 ± 4	13 ± 4	13 ± 5	16 ± 4	18 ± 4^c^	27 ± 4^d^
2b									
Norepinephrine (μg/kg/min)	–	–	0.01	0.03	0.05	0.1	0.2	0.3	0.3
LVEDA (cm^2^)	10.8 ± 1.0	9.3 ± 1.2^a^	10.0 ± 0.7	10.2 ± 1.4	10.4 ± 0.9^b^	10.5 ± 1.3	10.2 ± 1.2	9.9 ± 0.9^c^	10.5 ± 1.3
LVESA (cm^2^)	6.7 ± 1.1	5.4 ± 0.6^a^	5.8 ± 0.6	6.4 ± 1.0^b^	5.9 ± 0.8	5.9 ± 1.0	5.4 ± 1.2	5.1 ± 1.0^c^	5.4 ± 1.2^d^
IVC_Dia_ (cm)	1.0 ± 0.3	1.1 ± 0.2	1.1 ± 0.2	1.1 ± 0.2	1.2 ± 0.2	1.2 ± 0.2	1.2 ± 0.2	1.1 ± 0.2	0.9 ± 0.2^d^
dIVC (%)	12 ± 11	9 ± 7	6 ± 7	8 ± 9	7 ± 7	8 ± 5	7 ± 8	6 ± 4	5 ± 7
CVP (mmHg)	4 ± 4	8 ± 4^a^	8 ± 5	8 ± 4	8 ± 4	9 ± 5	9 ± 5	7 ± 4	6 ± 4^d^
Afterload (mmHg)	169 ± 42	131 ± 22^a^	140 ± 35	153 ± 33^b^	169 ± 32	181 ± 46	169 ± 44	162 ± 36^c^	168 ± 31
LVFAC (%)	39 ± 7	42 ± 8	42 ± 6	37 ± 9	43 ± 8	43 ± 7	48 ± 8	48 ± 10^c^	48 ± 8
MAP (mmHg)	72 ± 10	57 ± 9^a^	59 ± 7	62 ± 9	69 ± 12^b^	74 ± 19	81 ± 17	79 ± 14^c^	77 ± 12
CO (L/min)	1.9 ± 0.4	1.8 ± 0.3	1.9 ± 0.3	2.2 ± 0.8	2.3 ± 0.9	2.5 ± 1.0	2.7 ± 1.0	3.2 ± 0.9^c^	4.0 ± 0.7^d^
HR (bpm)	60 ± 5	64 ± 11	63 ± 14	68 ± 13	72 ± 19	74 ± 16	82 ± 22	87 ± 17	97 ± 22
LVEDP (mmHg)	9 ± 3	12 ± 3	11 ± 3	12 ± 3	12 ± 3	13 ± 3	14 ± 5	14 ± 6	11 ± 5^d^
PPV (%)	11 ± 4	10 ± 3	10 ± 3	9 ± 3	10 ± 3	10 ± 3	10 ± 4	11 ± 3	11 ± 4
PaO_2_ (kPa)	32 ± 4	12 ± 4^a^	12 ± 4	11 ± 4	11 ± 4	12 ± 5	11 ± 4	11 ± 6	27 ± 6^d^
2c									
Control	–	–	–	–	–	–	–	–	–
LVEDA (cm^2^)	12.2 ± 0.6	10.3 ± 1.3^a^	10.6 ± 1.3	10.4 ± 1.3	10.7 ± 1.3	10.4 ± 1.4	10.2 ± 1.5	10.4 ± 1.5	12.1 ± 1.0^d^
LVESA (cm^2^)	7.3 ± 1.2	6.1 ± 1.5	6.4 ± 1.0	6.1 ± 1.1	6.2 ± 1.5	5.8 ± 1.0	5.6 ± 1.2	5.9 ± 0.6	7.4 ± 1.0^d^
IVC_Dia_ (cm)	1.1 ± 0.3	1.1 ± 0.3	1.3 ± 0.3	1.1 ± 0.2	1.2 ± 0.3	1.2 ± 0.3	1.2 ± 0.3	1.1 ± 0.3	1.1 ± 0.2
dIVC (%)	8 ± 7	4 ± 6	7 ± 8	3 ± 5	5 ± 7	5 ± 6	2 ± 4	8 ± 7	14 ± 8^d^
CVP (mmHg)	3 ± 1	6 ± 1^a^	7 ± 2^b^	6 ± 1	7 ± 1	7 ± 2	7 ± 1	7 ± 1	5 ± 2
Afterload (mmHg)	180 ± 40	132 ± 58	138 ± 39	136 ± 26	122 ± 33	144 ± 37	120 ± 39	121 ± 23	144 ± 34^d^
LVFAC (%)	41 ± 9	44 ± 15	43 ± 6	41 ± 7	43 ± 9	45 ± 3	45 ± 5	43 ± 4	38 ± 7
MAP (mmHg)	80 ± 12	56 ± 12^a^	58 ± 8	58 ± 6	58 ± 6	57 ± 5	57 ± 7	57 ± 5	61 ± 7
CO (L/min)	2.5 ± 0.8	2.0 ± 0.5	2.1 ± 0.4	2.0 ± 0.3	2.1 ± 0.5	2.0 ± 0.4	1.9 ± 0.4	1.9 ± 0.3	2.2 ± 0.6
HR (bpm)	65 ± 9	58 ± 8	60 ± 9	61 ± 9	62 ± 10	59 ± 10	60 ± 9	58 ± 10	56 ± 8
LVEDP (mmHg)	7 ± 3	8 ± 4	8 ± 3	8 ± 2	10 ± 2	9 ± 4	8 ± 2	9 ± 3	7 ± 3
PPV (%)	14 ± 8	11 ± 3	10 ± 2	10 ± 3	10 ± 3	10 ± 1	11 ± 2	11 ± 1	11 ± 3
PaO_2_ (kPa)	32 ± 2	11 ± 3^a^	9 ± 3	14 ± 6	17 ± 7	15 ± 5	18 ± 7	18 ± 5^c^	31 ± 2^d^

The haemodynamic variables as mean ± standard deviation at baseline during 75 mL/kg pleural effusion and at recovery after pleurocentesis are depicted

*Abbreviations*: *LVEDA* left ventricular end-diastolic area, *LVESA* left ventricular end-systolic area, *IVCDia* expiratory inferior vena cava diameter, *dIVC* inferior vena cava distensibility, *CVP* central venous pressure, *Afterload* left ventricular afterload, *LVFAC* left ventricular fractional area change, *MAP* mean arterial pressure, *CO* cardiac output, *HR* heart rate, *LVEDP* left ventricular end-diastolic pressure, *PPV* pulse pressure variation, *Pa02* arterial partial pressure of oxygen

^a^Compared with baseline (*p* < 0.05)

^b^Compared with baseline (*p* > 0.05 for no difference)

^c^Difference over time from PLE (*p* < 0.05)

^d^Recovery values compared with maximum fluid load or norepinephrine infusion rate (*p* < 0.05)

### Model of pleural effusion

Preload, measured as LVEDA, decreased after PLE installation (*p* < 0.001), while LV end-diastolic pressure increased (*p* = 0.001). CVP increased simultaneously (*p* < 0.001). LV afterload, MAP, PaO_2_ and CO decreased significantly (*p* values ≤ 0.001). IVC diameter, IVC distensibility and pulse pressure variation were not significantly affected by PLE installation (*p* values ≥ 0.352; Table 1).

### Effects of fluid loading and norepinephrine infusion after installation of pleural effusion

#### Preload

LVEDA increased during both fluid infusion and increments in norepinephrine infusion rates (*p* values < 0.001; Table 2 (2a, 2b)). Meanwhile, fluid loading increased LV end-diastolic pressure and decreased pulse pressure variation (*p* values < 0.001). At a fluid load of 20 mL/kg and a norepinephrine infusion rate of 0.05 μg/kg/min, LVEDA was restored (*p* values ≥ 0.061). Numerical baseline values were not reached in the norepinephrine group, and LVEDA decreased at subsequent higher infusion rates.

Evacuation of PLE increased the numerical values of LVEDA in all groups, although only statistically significantly so in the control group. In contrast, only LV end-diastolic pressure decreased after evacuation in the intervention groups (*p* values < 0.001).

#### Afterload

LV afterload increased over time in both intervention groups (*p* values < 0.001; Table 2 (2a, 2b)). LV afterload was normalised at fluid loads ≥ 20 mL/kg (*p* values ≥ 0.944) and at norepinephrine infusion rates ≥ 0.03 μg/kg/min (*p* values ≥ 0.306). Evacuation of PLE did not affect LV afterload significantly in any group (*p* values ≥ 0.195).

#### Contractility

LV fractional area change was unaffected in the fluid loading group (*p* = 0.903; Table 2 (2a)). After an initial decreasing trend, LV fractional area change increased at norepinephrine infusion rates > 0.03 μg/kg/min (*p* values > 0.002; Table 2 (2b)). LV fractional area change decreased in the fluid loading group only (*p* = 0.022) when PLE was evacuated.

#### Inferior vena cava and pulse pressure variations

IVC diameter increased in both the fluid loading and the control group (*p* values ≤ 0.048), but remained unchanged in the norepinephrine group (*p* = 0.931; Table 2 (2a–2c)). Fluctuations of mean values were within 2 mm.

IVC distensibility was unaffected in all groups except for an increase after PLE evacuation in the control group (*p* = 0.002).

PPV decreased in a dose-dependent manner in the fluid loading group only (*p* < 0.001). Likewise, CVP increased only during fluid loading (*p* < 0.001), but decreased after PLE evacuation in both intervention groups (*p* values < 0.001). CVP was unaffected in the control group (*p* = 0.115).

#### Systemic blood pressure

Fluid loading and incremental norepinephrine infusion rates increased MAP (*p* values < 0.001; Table 2 (2a, 2b)). MAP was restored after a fluid load of 20 mL/kg and at a norepinephrine infusion rate of 0.05 μg/kg/min (*p* values ≥ 0.416). No significant change in MAP was observed in any of the groups after evacuation (*p* values ≥ 0.205).

#### Variability

Mean intra-observer variability for all echocardiographic endpoints was − 0.4% (95% limits of agreement − 14.5–13.6%) (95% confidence interval − 0.9–0.0%) and mean inter-observer variability was 1.1% (95% limits of agreement − 14.8–17.0%) (95% confidence interval 0.6–1.5%).

## Discussion

Installation of pleural effusion decreased preload and markers of global circulation. These changes were effectively restored with both fluid loading and infusion of norepinephrine.

### Fluid loading

**Fig. 2:**
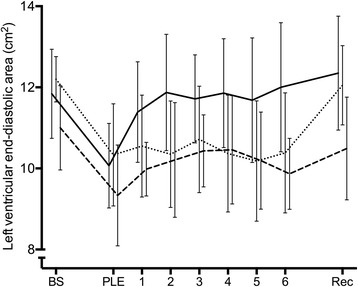
Left ventricular preload. Graph showing left ventricular end-diastolic area at baseline (BS), after pleural effusion installation (PLE), during the intervals of incremental intervention (1, 2, 3, 4, 5 and 6) corresponding to: fluid loading (20, 40, 60, 80, 100 and 120 mL/kg) (full line), norepinephrine infusion (0.01, 0.03, 0.05, 0.1, 0.2 and 0.3 μg/kg/min) (dashed line) and placebo (dotted line), and finally at recovery after pleurocentesis (Rec). Left ventricular end-diastolic area decreased due to pleural effusion, which was normalised by fluid loading and norepinephrine infusion. The effect subsided at higher infusion rates of norepinephrine. Subsequent evacuation only caused an additional increase in left ventricular end-diastolic area in the control group

Moderate amounts of fluid loading (20 mL/kg) restored LVEDA (Fig. 2) and normalised MAP, CO and pulse pressure variation. As systemic blood pressure was quickly restored, this treatment clearly involves a risk of misdiagnosis. Hence, PLE mimics hypovolaemic or distributive shock both in its clinical appearance and the effects of fluid loading. This may hamper diagnosis of PLE or falsely reduce the perceived clinical significance of a known PLE. However, the immediate rise in CVP to supranormal values (Table 2 (2a)) following fluid loading testified to the volume overload induced by fluid loading, potentially subjecting recipients to the harmful effects of compromised organ microcirculation [21, 22].

### Norepinephrine infusion

Relatively low infusion rates of norepinephrine restored LVEDA, CO, MAP and LV afterload (Fig. 2), hence nullifying the haemodynamic effects of PLE. In parallel to fluid loading, haemodynamic restoration was easily accomplished with a first-line treatment for hypotension, although still not treating the underlying cause.

First, α_1_-stimulation contracts peripheral, systemic vasculature, and the resulting increase in LV afterload may to some extend impede LV ejection and subsequently increase LVEDA [23]. Second, stimulation of myocardial β_1_-receptors enhances contractility and maintains heart rate [24]. Third, the biphasic effect of norepinephrine may be explained by its receptor affinity. First, the splanchnic and hepatic vessel beds act as a reservoir of blood (unstressed volume), and stimulation of α_1_, α_2_ and β_2_-receptors in these vessel beds, and in turn increases the stressed blood volume, venous return and consequently LVEDA [25, 26].

Norepinephrine increased LVEDA from 9.3 ± 1.2 to 10.5 ± 1.3 cm^2^ from installation of pleural effusion to a norepinephrine dose of 0.1 μg/kg/min despite an approximate 10% increase in HR. LV fractional area change was constant. As CO increased by 39% (1.8 ± 0.3 to 2.5 ± 1.0 L/min) whereas MAP increased by a comparable 30% (57 ± 9 to 74 ± 19 mmHg, see Fig. 2b), systemic vascular resistance must have changed minimally (MAP = CO × systemic vascular resistance). Therefore, the effect on LVEDA was primarily mediated by an increase in venous return. At high doses of norepinephrine (> 0.1 μg/kg/min), LVEDA decreased; we attribute this to myocardial β_1_-receptor stimulation as LV fractional area change increased concomitantly.

### Measures of inferior vena cava

The marked decrease in LV preload and doubling of CVP after installation of PLE were not mirrored in measures of IVC dimensions (Table 1). Extensive fluid loading and an accompanying substantial increase in CVP did not affect the respiratory variation of the IVC, whereas the expiratory diameter of the IVC increased. However, the increase of 2 mm was negligible and close to practical measurement error [27]. Hence, our findings do not support IVC measurements as reliable indices of CVP in the presence of PLE, although these are related [28, 29]. Likewise, the initial increase and subsequent levelling out in CO caused by fluid loading was not reflected in changes in IVC respiratory variations, de-emphasising IVC dynamics as a measure of preload responsiveness when PLE is present [30, 31].

### Installation of pleural effusion

This animal model confirmed the haemodynamic effects of PLE including an increase in CVP and concomitant decreases in arterial blood pressure, PaO_2_ and CO [4, 5, 7, 16] (Table 1). LV fractional area change showed an increasing trend, but this was not a consequence of a higher inotropic state, but instead due to a reduced preload and a decrease in LV transmural pressure as LV end-diastolic pressure increased.

Together with the decreases in MAP and CO, the increases in LV end-diastolic pressure and CVP testify to the pathophysiological effect of pleural effusion. As described in a previous study [6], pleural effusion likely decreased biventricular transmural pressures and, hence, effective filling pressures and ventricular volumes. PaO_2_ was reduced markedly with pleural effusion, but did not reach sub-normal levels so we find it unlikely that PaO_2_ levels influenced haemodynamic parameters.

PLE did not lead to changes in pulse pressure variation, though an increase was expected. However, a study with a comparable PLE intervention also detected only slight increases in pulse pressure variation [8]. While not addressing pulse pressure variations’ fluid responsiveness prediction abilities in this study and merely addressing physiology, we speculate that the significant PLE-induced changes in lung mechanics [8] may reduce pressure transmission to the pleural space during ventilation and, as such, may reduce the effective preload changes responsible for pulse pressure variation. Therefore, pulse pressure variation should probably be interpreted with caution when PLE is present. Apart from the effect of PLE, pulse pressure variation behaved as expected by declining in the fluid group and not changing in the two other groups.

### Evacuation of pleural effusion

Evacuation of PLE altered most endpoints in the control group significantly or with a convincing trend (Table 2 (2b)). These effects were less obvious in the fluid loading group and in the norepinephrine group as numerical changes were virtually absent.

PaO_2_ increased markedly in all groups after evacuation regardless of intervention. Altogether, these observations favour early detection and drainage of PLE [3], as fluid load or infusion of norepinephrine have considerable side effects.

### Clinical implications

This study confirms the profound effects pleural effusion may elicit on key haemodynamic variables. The decrease in arterial pressure and CO together with a rise in CVP, seen with pleural effusion, is synonymous with cardiac failure or pulmonary embolism to many clinicians and, seen together, emphasises the potential benefits of ultrasonographic visualisation of the heart and lungs.

Nevertheless, both fluid loading and infusion of norepinephrine effectively reserved the haemodynamic changes of pleural effusion. This underscores the value of these treatments as first-line options, but also reveals a risk of misdiagnosis, as physicians may attribute PLE-induced hypotension to hypovolaemia or vasodilatation, when either fluid- or vasopressor therapy prove effective.

### Limitations

This study was conducted in an experimental model of young and healthy pigs, precluding direct extrapolation to patients with PLE, who often suffer from significant comorbidities. Additionally, PLE was induced rapidly, whereas patients often accumulate PLE slowly. The haemodynamic implications of the latter have not been described. The protocol comprised persistent and large amounts of fluid and norepinephrine, which might not resemble clinical practise. This was chosen as to evaluate the effect, or the lack of it, during overtreatment.

Also, the amount of pleural fluid installed was substantial when considering the size of the piglets. This amount was chosen from a previous study to ensure a haemodynamic effect of pleural effusion in physiologically intact animals [7]. The relationship between pleural effusion volume and haemodynamic effect in critically ill humans has not been described systematically.

## Conclusions

PLE significantly reduced LV preload, MAP, PaO_2_ and CO despite increasing absolute cardiac filling pressures. Both fluid loading and low norepinephrine infusion rates reverted this preload decrease and normalised most other frequently measured haemodynamic parameters. Interestingly, extensive fluid loading and high-dose norepinephrine infusion prevented the haemodynamically beneficial effects of pleuracentesis. In addition, this study elucidated the risk of attributing PLE-induced circulatory compromise to hypovolaemia or vasodilatation resulting in further administration of volume or inoconstriction without addressing the underlying cause.

## Abbreviations

Afterload: Left ventricular afterload
CO: Cardiac output
CVP: Central venous pressure
HR: Heart rate
IVC: Inferior vena cava
dIVC: Inferior vena cava distensibility
IVC_Dia_: Expiratory inferior vena cava diameter
LV: Left ventricle
LVEDA: Left ventricular end-diastolic area
LVEDP: Left ventricular end-diastolic pressure
LVESA: Left ventricular end-systolic area
LVFAC: Left ventricular fractional area change
MAP: Mean arterial pressure
PaO_2_: Arterial partial pressure of oxygen
PPV: Pulse pressure variation
